# Axial Compression Properties of Recycled Concrete-Filled Circular Steel Tubular Column Subject to Corrosion

**DOI:** 10.3390/ma18174003

**Published:** 2025-08-27

**Authors:** Dongxia Hu, Jin Wu, Zhe Feng, Renming Liu, Shefeng Guo, Liqiang Liu

**Affiliations:** 1 College of Civil Aviation, Nanjing University of Aeronautics and Astronautics, No. 29, Yudao St., Naniing 211106, China; hudongxia_xj@163.com (D.H.); 14799747612@163.com (Z.F.); gsf20240411@163.com (S.G.); 2 School of Road and Bridge Engineering, Xinjiang Vocational and Technical College of Communications, No. 478, Yongshun St., Urumchi 831401, China; 3 Xinjiang Communications Construction Group Co., Ltd., No. 840 Wuchangfu Road, Urumchi 830006, China; 18199303479@163.com; 4 Xinjiang Road and Bridge South Xinjiang Engineering and Construction Co., No. 74, Culture Road, Kashgar 844000, China; jiedishisheng@163.com

**Keywords:** circular recycled concrete-filled steel tubular short columns, chloride ion corrosion, axial compression performance, ultimate load capacity, mechanical properties

## Abstract

In order to investigate the change in the axial compression performance of circular recycled concrete-filled steel tubular short columns under chloride ion corrosion, 24 circular recycled concrete-filled steel tubular (RCFST) short columns and 12 circular natural concrete-filled steel tubular (NCFST) short columns for axial compression tests after being subjected to different corrosion degrees were designed. The experimental parameters include the corrosion degree (0, 2, 4, 6, 8, 10, 12, 14%) and the recycled concrete replacement rate (0, 100%). The experimental results show that the damage mode of the specimen after corrosion is localized buckling deformation of the steel tube. Due to the good confinement effect of the steel tube, the internal concrete was crushed only at the localized buckling part of the steel tube. The stiffness and ductility decreased significantly with increasing corrosion degree. As the corrosion degree increased from 0 to 14%, the stiffness of the circular RCFST short columns decreased by approximately 36.3%, and the ductility dropped by around 23.3%. And the corrosion resistance of the circular RCFST short column was worse than that of the circular NCFST short column. Based on the experimental results, the ultimate load capacity calculation model of the circular concrete-filled steel tubular short column is proposed.

## 1. Introduction

In recent years, recycled aggregate concrete has become a research hotspot. Recycled aggregate concrete refers to a new environmentally friendly building material that is produced by crushing and screening waste concrete into recycled aggregates, which are then used to replace natural aggregates in a certain proportion. The emergence of recycled concrete not only realizes the utilization of construction waste resources but also helps alleviate the problem of rising prices of natural sand and gravel. Numerous scholars have studied the mechanical properties of recycled aggregate concrete, and it has been found that, compared to natural concrete, the strength of recycled aggregate concrete is reduced [1]. However, it has also been proven that recycled aggregate concrete can be used in engineering practice [2].

Concrete-filled tubular steel is a special composite structure that utilizes the effective confinement of steel tubes on the internal concrete, allowing the concrete to be subjected to tri-axial stress and thus providing a higher load-bearing capacity and deformation performance. It has been shown that RCFST has a much higher energy absorption capacity than recycled concrete [3]. Additionally, the presence of concrete effectively prevents the buckling of the steel tubes and fully utilizes the advantages of the steel tubes and recycled concrete [4]. Compared to conventional steel tube reinforced concrete structures, steel tube recycled concrete exhibits similar mechanical properties and failure modes, and it possesses excellent load-bearing capacity. Therefore, it has broad application prospects in various fields [5]. Zhao [6] studied the performance of steel tube columns filled with recycled concrete instead of natural concrete and found that recycled concrete-filled tubular steel is a reasonable and effective type of utilization.

Many researchers have conducted systematic studies on the axial compression performance of recycled circular concrete-filled tubular steel columns, both circular and rectangular in shape. There are numerous factors that influence the axial compression performance of RCFST columns, such as the thickness and slenderness ratio of the steel tube, the replacement ratio of the recycled aggregates, and the core concrete strength. Zhang [7] investigated the effect of different coarse aggregate replacement rates on the bearing capacity of RCFST columns through experiments and found that the bearing capacity tends to decrease with the increase in the replacement rate. Wang [8] also obtained the same conclusion and proposed the stress–strain relationship between the steel tube and the core recycled concrete. Geng [9] established a long-term loading finite element model based on experiments and found that the incorporation of recycled aggregates increases the long-term performance of the RCFST columns by more than 40%. Yang [10] found that the working mechanism and failure mode of rectangular RCFST columns are similar to those of natural circular concrete-filled steel tubular (NCFST) columns, but the addition of recycled aggregates leads to an earlier occurrence of local buckling of the steel tube. The negative impact of an increased replacement ratio on the axial stiffness of rectangular RCFST columns is greater than its effect on load-bearing capacity. The stiffness of RCFST with 100% recycled aggregate replacement was reduced by about 20% compared to NCFST. In addition, Zhang [11] and Ma [12] separately studied the axial compression performance of fiber-reinforced RCFST columns and found that the addition of fibers could successfully retard the development of concrete cracks and improve the cooperative deformation capacity of steel tubes and concrete.

With the development of industrialization, the corrosion problem of chloride salts has had a serious impact on the safety and use of building structures. Concrete-filled steel tubular structures are subject to chloride ion erosion in marine, coastal, and acid rain and various salinized land environments. The corrosion of steel tubes can result in rusting, which not only diminishes the modulus of elasticity and strength of the material but also leads to a reduction in the cross-sectional area and deterioration of material properties. And this degradation affects the confinement efficacy of the steel tube on the surrounding concrete. This phenomenon may ultimately lead to a significant decline in the overall structural performance of circular concrete-filled steel tubular (CFST) members, potentially culminating in catastrophic events such as structural collapse and thereby posing a severe threat to the safety and integrity of the structure. Consequently, the investigation into the axial compression performance and the development of a design methodology for CFST members subjected to chloride salt corrosion holds significant engineering practical value. Han [13] and Hua [14] conducted a comprehensive investigation into the mechanical properties of square CFST short columns exposed to chloride corrosion. Their findings revealed that a high degree of corrosion had a pronounced effect on the load-bearing capacity and ductility of the specimens, leading to significant reductions in both properties. Yang [15] established a finite element model for random corrosion of CFST columns and, compared with the experimental results, found that the constraint effect between the steel tube and concrete after corrosion was related to the mass loss rate but not to the depth of corrosion. To better approximate the engineering reality, Li [16] investigated the impact of regional corrosion on the axial compressive performance of CFST (concrete-filled steel tube) columns. The degree of regional corrosion was quantified using the thickness corrosion ratio and height corrosion ratio. As the degree of corrosion deepened, the axial compressive stiffness and bearing capacity of the specimens gradually decreased.

Despite the significant enhancement in the load-bearing capacity offered via RCFST columns when compared to recycled concrete structures, the inherent poor corrosion resistance of the steel poses a critical challenge. Consequently, a study of the corrosion resistance of RCFST columns is imperative for practical application. Huang [17] and Chen [18] separately studied the eccentric and axial compression performance of RCFST columns after acid rain corrosion. Both studies used the method of reducing the wall thickness to calculate the load-bearing capacity and achieved good results. Hui [19] investigated the effects of different wall thicknesses of steel tube and different corrosion degrees on the axial compressive properties of high-strength RCFST columns after chloride corrosion, and it was found that the bearing capacity of the specimen decreased with the increase in corrosion, and increasing the wall thickness of the steel tube could effectively improve the bearing capacity.

Under long-term loading, the durability of an RCFST column is crucial for its quality and service life. Corrosion resistance is an important indicator to evaluate the durability of RCFST. Therefore, conducting in-depth research on the performance of RCFST columns after corrosion is essential to ensure structural safety during service.

There is a greater amount of research available on the performance of NCFST columns after corrosion, while studies on RCFST columns are not yet well developed. The majority of studies on RCFST columns have concentrated on their performance after exposure to acid rain corrosion, with a comparatively limited scope devoted to chloride salt corrosion. Additionally, there is a larger body of research on rectangular steel tubular concrete columns and a relatively smaller body of research on round steel tubular concrete columns. Furthermore, research on the use of seamless steel tubes is also relatively less. Therefore, the aim of this study is to investigate the mechanical properties of round seamless steel tubular recycled concrete short columns after experiencing different degrees of corrosion. In total, 36 RCFST column specimens were subjected to axial compression tests. Based on the test results, the ultimate axial bearing capacity of the corroded columns was studied, and a formula for calculating the axial compressive capacity was derived based on relevant specifications.

## 2. Materials and Methods

### 2.1. Specimen Design

**Figure 1:**
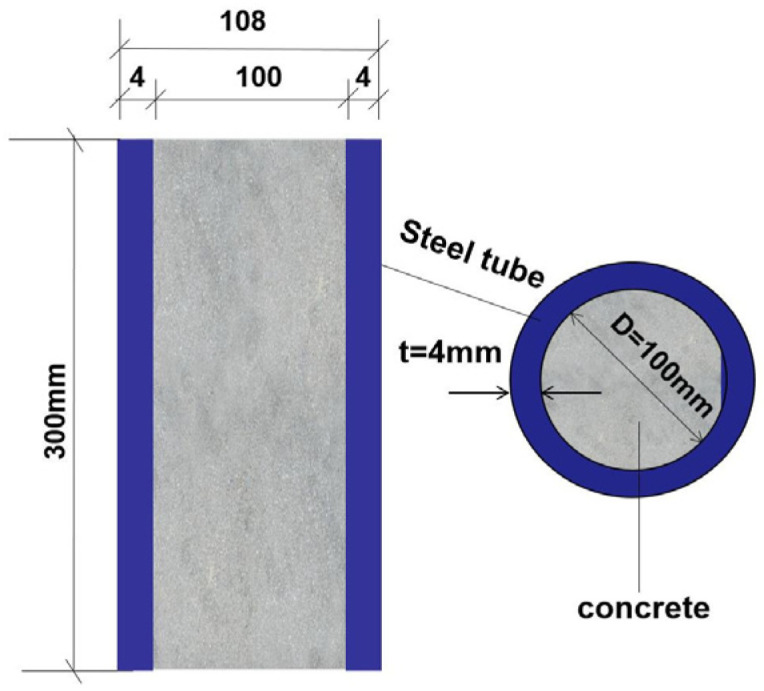
Detailed dimensions of the specimens.

A total of 36 recycled concrete-filled circular steel tube columns with a strength grade of C30 were designed and cast. The test parameters mainly included the replacement rate of recycled coarse aggregate (0, 100%) and the corrosion degree (0, 2, 4, 6, 8, 10, 12, 14%). The specific dimensions of the specimens are shown in Table 1 and Figure 1.

### 2.2. Materials

**Figure 2:**
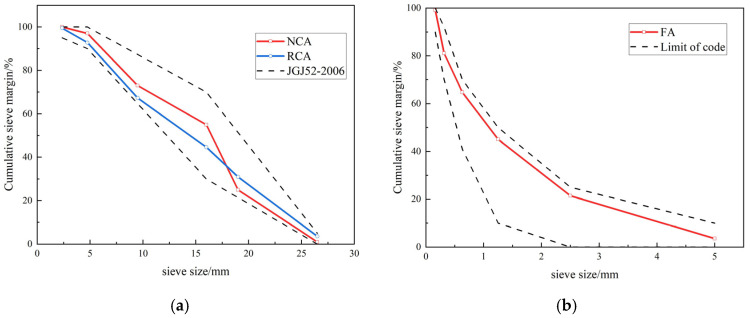
Particle gradation of aggregate: (**a**) coarse aggregate; (**b**) fine aggregate.

Natural concrete and recycled concrete both used P.O 42.5 ordinary Portland cement and natural river sand with a fineness modulus of 2.43. The water used was the tap water from Nanjing City, China. The coarse aggregates all have a continuous gradation of 4.75 to 26.5 mm, and the specific particle gradation is shown in Figure 2. The limit of the code in the figure was obtained from JGJ52-2006 [20].

In addition, the basic properties of recycled coarse aggregates are shown in Table 2. According to the GB/T 25177-2010 [21], the RCA used in the test is class II aggregates. The designed strength grade for concrete specimens was C30 [22], and the average measured compressive strength of natural concrete and recycled concrete cube specimens are 33.1 and 29.9 MPa, respectively. And the detailed mix proportion was presented in Table 3. The steel tube was made of No. 20 steel. A seamless 20 steel tube is used for the outward tube.

### 2.3. Test Procedure

#### 2.3.1. Corrosion Test

**Figure 3:**
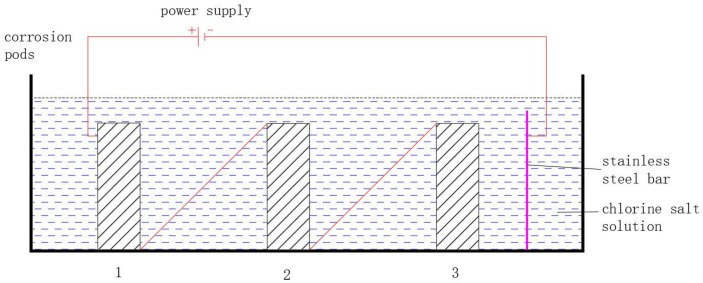
The corrosion test setup.

To simulate actual situations within a relatively short period of time, the method of indoor accelerated electrochemical corrosion testing was used. The corrosion solution was a 3% concentration chloride salt solution, which was obtained by adding industrial-grade chloride salt to distilled water at a temperature of 25 ± 2 °C. The corrosion device diagram is shown in Figure 3. The specimens are connected to each other in series connections. Enough corrosion solution is poured in to ensure that the specimen is fully immersed in the solution. The positive terminal of the regulated power supply is directly connected to the specimen, and the negative terminal is connected to the stainless steel rod. The duration of electrification is determined based on Faraday’s law.

In order to accurately measure the corrosion degree of the steel tube, it is necessary to remove the core concrete after the experiment ends and then conduct measurements. The specific method should be operated in accordance with the provisions of GB/T50082-2009 [23]. The corrosion degree of the steel tube can be calculated using the following formula:

(1) $$\gamma =\frac{\Delta m}{m_{0}}\times 100\%=\frac{m_{0}-m_{i}}{m_{0}}$$

where the following applies: γ refers to the corrosion degree; Δm refers to the mass loss; $m_{0}$ refers to the mass of the specimen before corrosion; $m_{i}$ refers to the mass of the specimen after corrosion.

**Figure 4:**
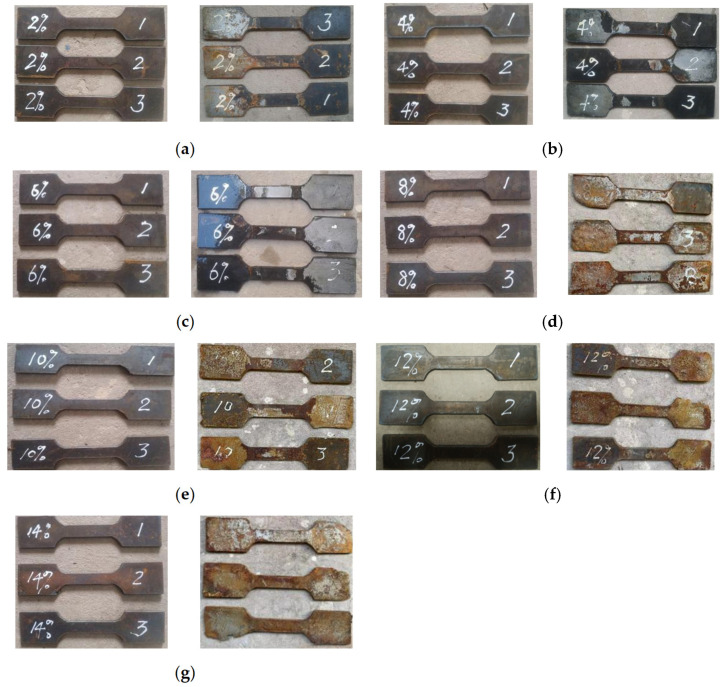
The comparison diagram of 4 mm-thick tensile test specimens before and after different degrees of corrosion: (**a**) 2%; (**b**) 4%; (**c**) 6%; (**d**) 8%; (**e**) 10%; (**f**) 12%; (**g**) 14%.

**Figure 5:**
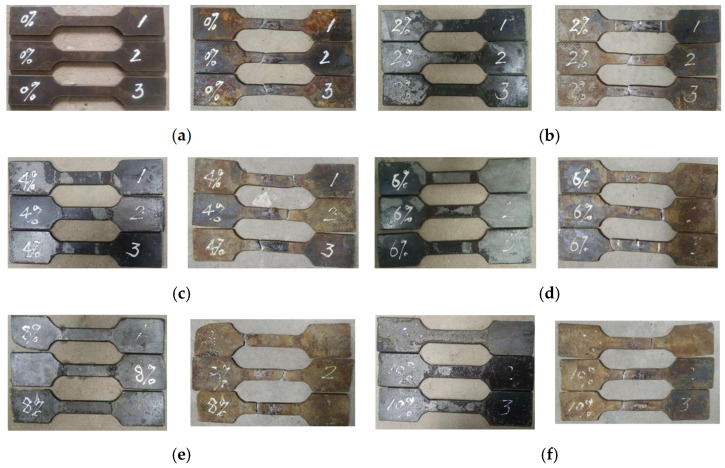

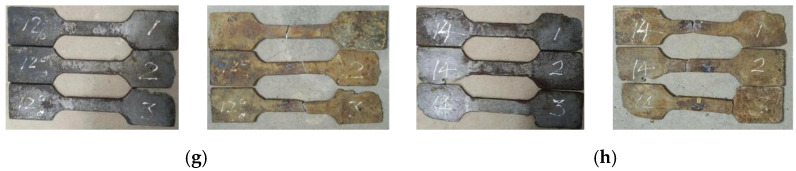
The comparison of 4 mm-thick tensile specimens before and after stretching at different degrees of corrosion: (**a**) 0%; (**b**) 2%; (**c**) 4%; (**d**) 6%; (**e**) 8%; (**f**) 10%; (**g**) 12%; (**h**) 14%.

Corresponding tensile specimens were prepared to investigate the mechanical properties of the steel tube at different degrees of corrosion. The test results of tensile specimens are shown in Table 4. Figure 4 shows the changes in tensile specimens with different degrees of corrosion before and after corrosion. It can be seen that as the degree of corrosion increases, the degree of rusting on the surface of the tensile specimens becomes more severe, and this pattern becomes more obvious when the degree of corrosion exceeds 6%. Figure 5 shows a comparison of tensile specimens before and after stretching at different degrees of corrosion. Before the tensile test, the tensile specimens were rust-removed. However, since they were left for a period of time after rust removal, there were slight traces of rust on the surface of the specimens during the tensile test.

**Figure 6:**
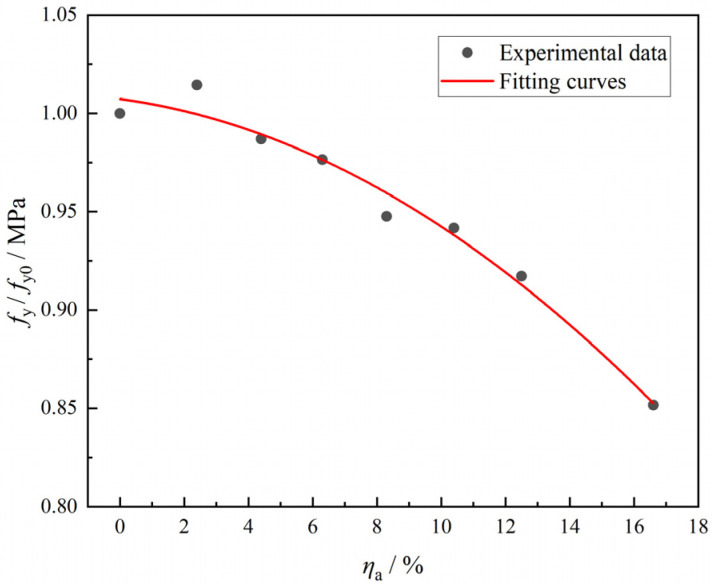
Relationship between yield strength loss and corrosion rate of steel tube.

The quantitative relationship between the ratio of yield strength (yield strength after corrosion of the specimen $f_{y}$/the yield strength of the uncorroded specimen $f_{y0}$) of corroded steel and the corrosion degree (*η*_a_) can be determined through regression analysis using the least squares method. The fitting curve is shown in Figure 6, and the regression result is as follows:

(2) $$f_{y}/f_{y0}=(-4.29{\eta_{a}}^{2}-0.22\eta_{a}+1)\;\;\;\;\;R^{2}=0.97$$

#### 2.3.2. Axial Compression Test

**Figure 7:**
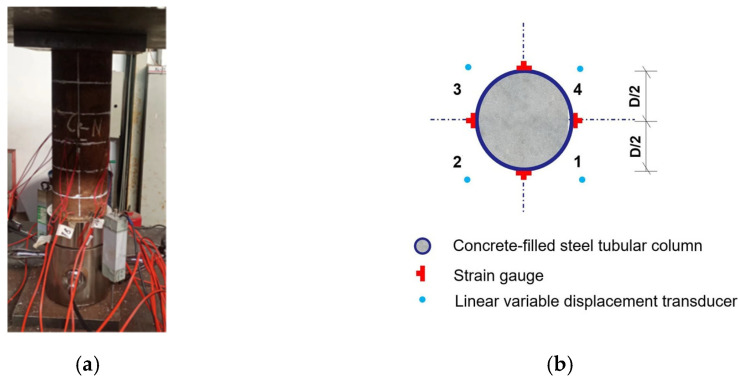
Detailed dimensions of the specimens: (**a**) test setup; (**b**) strain gauge attachment diagram.

Loading protocol for axial compression test of C30 concrete-filled 15# seamless circular steel tube columns (outer diameter 108 mm, wall thickness 4 mm, length 300 mm): The test specimens are concrete-filled steel tube columns with the above parameters. The 500 t hydraulic testing machine in the Structural Laboratory of Nanjing University of Aeronautics and Astronautics is used for graded monotonic static loading. To verify the proper functioning of specimens and testing instruments, a pre-loading process was carried out prior to the start of the experiment. After the pre-loading was completed, the formal loading commenced, utilizing a step-by-step loading method. During the elastic stage, each load increment was set to 10% of the estimated ultimate load. Each load level was maintained for 2 min, during which axial/radial displacements, steel tube strains, and specimen behaviors at each load step were recorded simultaneously. The test was terminated when the load dropped to 80% of the ultimate load or obvious instability and severe buckling of the steel tube occurred. The test setup is shown in Figure 7. In order to measure the longitudinal and transverse strains of the RCFCST column during compression of the specimen, eight strain gauges were evenly pasted in the ring direction in the middle of the column height, including four longitudinal and four transverse strain gauges, as shown in Figure 7. Two linear variable differential transformers (LVDTs) with a range of 100 mm and an accuracy of 1 mm were uniformly arranged along the circumference of the specimen to measure the longitudinal deformation of the specimen.

## 3. Results and Discussion

### 3.1. Failure Mode

The deformation of the specimen increases slowly at the beginning of the loading when it is in the elastic phase. As the load increases, the deformation accelerates, and the specimen begins to enter the plastic phase. At about 55% of the ultimate load, unidirectional oblique shear slip lines begin to appear on the surface of the steel pipe. With the further increase in the axial load, the number of shear slip lines gradually increases, and the rust powder on the surface of the steel pipe is continuously dislodged. When the load reaches about 85% of the ultimate load, the internal concrete is squeezed, and the surface of the specimen begins to buckle and deform. After reaching the ultimate load, the buckling deformation of the specimen surface increases rapidly, and the number of buckling deformation surfaces also increases. The failure process of the RCFST column is similar to that of the NCFST column. There is no significant difference in the failure process of RCFST before and after corrosion, which is consistent with the results of other scholars’ research on uncorroded RCFST columns [5,6,7].

The damage process of the specimens was approximately the same. Local buckling occurred earlier in the RCFST short columns than in the NCFST short columns, which was mainly due to the incorporation of recycled coarse aggregate. The strength of recycled concrete is lower than that of normal concrete for the same water–cement ratio; thus, the concrete has less of a filling effect on the steel tube. As the corrosion degree increased, the sound of concrete being crushed was more pronounced, which occurred since the increase in the corrosion degree caused the outward steel tube to restrain the concrete less.

**Figure 8:**
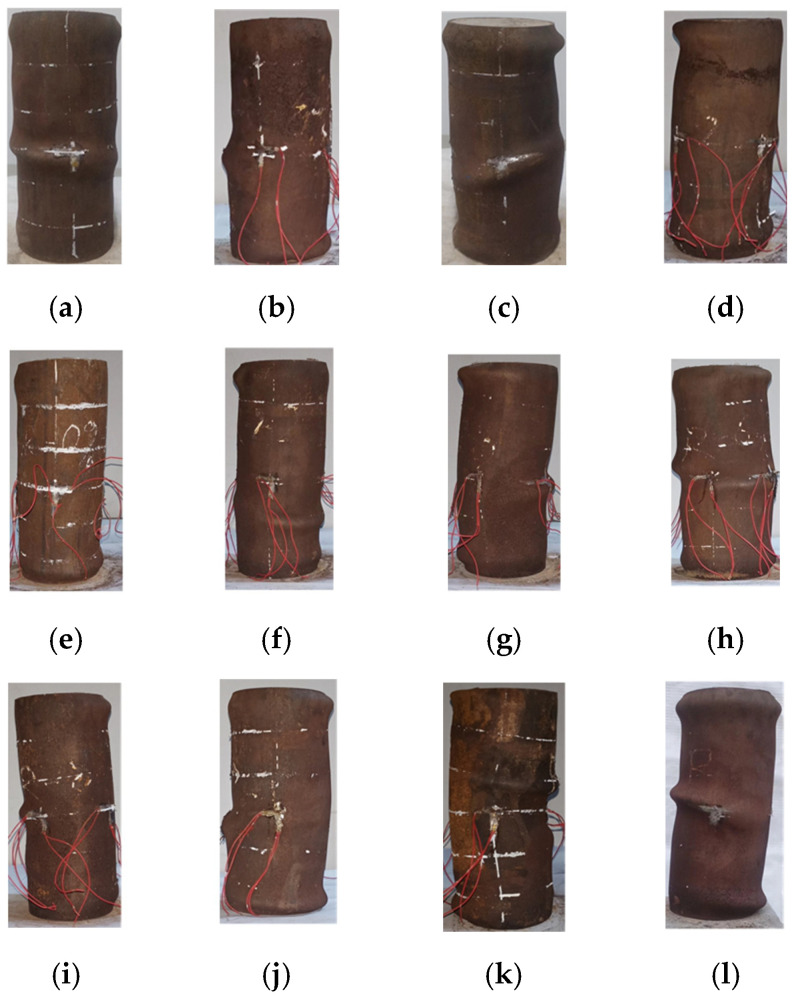
Failure mode of the specimens: (**a**) NCFST-0-2; (**b**) NCFST-2-2; (**c**) NCFST-6-2; (**d**) NCFST-12-2; (**e**) RCFST-0-2; (**f**) RCFST-2-2; (**g**) RCFST-4-2; (**h**) RCFST-6-2; (**i**) RCFST-8-2; (**j**) RCFST-10-2; (**k**) RCFST-12-2; (**l**) RCFST-14-2.

The damage pattern of the corroded specimens was similar to that of the uncorroded specimens. From Figure 8, it can be observed that the specimens in this experiment are mostly damaged in the form of a waist drum. This is mainly due to the stronger restraining effect of the steel tube on the internal concrete. In addition, regardless of the type of concrete and the degree of corrosion, the specimen will buckle outward [24]. Most of the specimens showed buckling deformation in the upper or lower part due to the end effect. With the increasing load, single or multiple severe buckling deformations appeared in the middle of the specimens. The failure patterns of the corroded NCFST and RCFST are similar to those of the uncorroded specimens, with buckling at the ends and middle. The mode of destruction is consistent with the results obtained by other scholars [5,6,7]. The buckling of the corroded specimen is more pronounced in the middle of the specimen, which may be due to the geometric defects of the steel tube itself.

**Figure 9:**
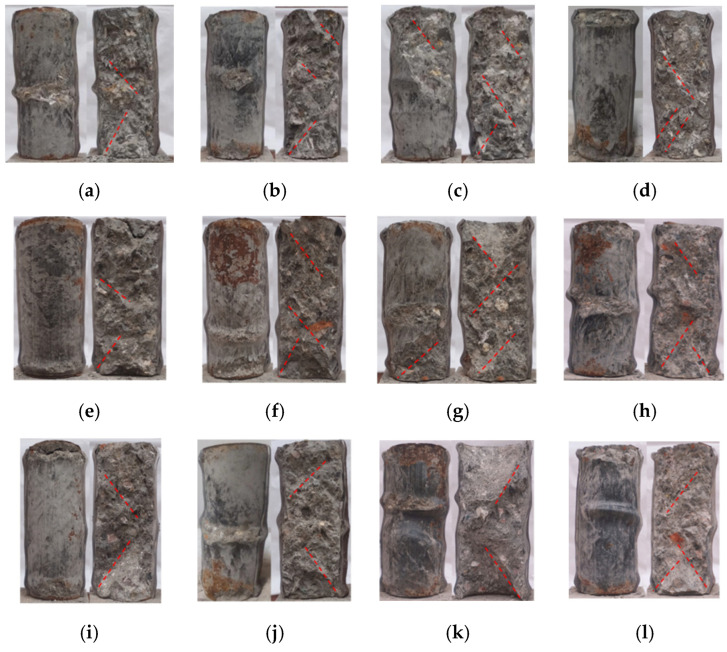
Failure mode of the core concrete: (**a**) NCFST-0-2; (**b**) NCFST-2-2; (**c**) NCFST-6-2; (**d**) NCFST-12-2; (**e**) RCFST-0-2; (**f**) RCFST-2-2; (**g**) RCFST-4-2; (**h**) RCFST-6-2; (**i**) RCFST-8-2; (**j**) RCFST-10-2; (**k**) RCFST-12-2; (**l**) RCFST-14-2.

To observe the breakage of the core concrete and the interaction between the external steel tube and the core concrete, the tested specimen was dissected along a certain symmetrical plane of the steel tube to expose its core concrete. The deformation location of the core concrete is the same as the deformed part of the steel tube, as shown in Figure 9. Only the core concrete at the site of local buckling deformation of the steel tube is crushed, and the concrete surface has almost no cracks [25]. This is mainly due to the better restraining effect of the steel tube, and the plasticity of the core concrete is greatly improved under the restraining effect of the steel tube. To further observe the damage pattern of the concrete, the core concrete was dissected along the bottom surface of the core concrete in any diameter direction. From Figure 8, it can be seen that the typical damage of the specimen is shear failure, with diagonal shear cracks appearing in the inner core concrete (indicated by the red dashed line in the Figure 9), and the angle between the cracks and the horizontal line is about 60°. The internal concrete failure mode of the RCFST column and the NCFST column is basically the same [5]. The shear damage is more pronounced as the corrosion degree increases. This is attributed to the weakened confinement of the concrete by the corroded external steel tube. The shear failure of concrete within NCFST and RCFST short columns is predominantly associated with the inherent characteristics of concrete materials and the prevailing stress state. Concrete inherently exhibits significantly lower shear resistance compared to its compressive capacity. Moreover, it contains natural internal defects, such as microcracks and pores. Under high-pressure conditions, these defects are prone to expansion and interconnection, ultimately leading to the formation of a shear failure plane. Meanwhile, owing to the short column’s relatively small height-to-diameter ratio, shear deformation becomes more pronounced under axial compression. Additionally, at the interface between the steel tube and the concrete, local shear stress concentration may occur due to inadequate bond performance or an uneven confinement effect. This, in turn, further exacerbates the risk of shear failure in the concrete.

### 3.2. Load-Axial Displacement Curves

**Figure 10:**
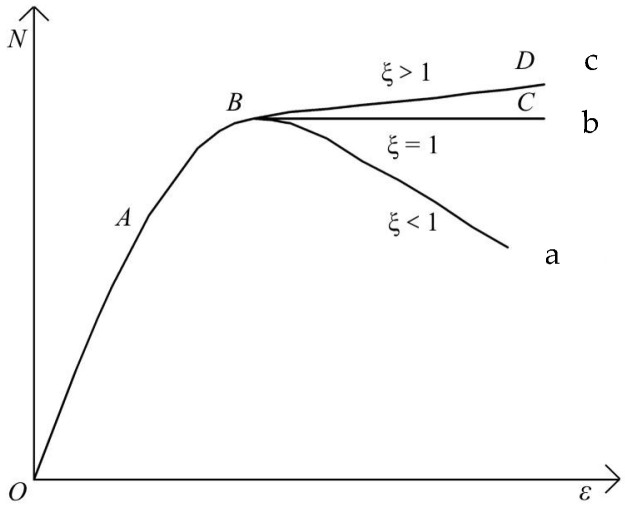
The load–displacement curve type of NCFST.

The constraint coefficient can better reflect the constraint effect of steel tubes on concrete. There are three main situations in the load–displacement curves of axially compressed circular concrete-filled steel tubular (CFST) short columns, as shown in Figure 10 [5].

The steel content of a CFST column in the actual project is usually in the range of 6~18%, and the constraint coefficient is usually greater than or equal to 1.0. Therefore, the constraint coefficient of >1.0 is designed in this experiment to be closer to the actual project (curve c). The load–displacement curves of CFST columns with a constraint coefficient > 1.0 have the following three main stages.

The OA is the elastic phase, the load-displacement relationship curve is close to a straight line. Point A is about 70~80% of the ultimate load. At this stage, the steel tube and concrete are pressurized at the same time, but because the lateral deformation of the steel tube is larger than that of the concrete, the steel tube does not have a restraining effect on the concrete at this stage.

The AB is the elastic–plastic stage. In this stage, the load–displacement relationship curve deviates from the straight line. With the increase in axial force, when the transverse deformation of concrete exceeds the steel tube, the steel tube plays a restraining effect on the concrete, and the concrete enters the three-way stress state.

The BD section is the strengthening stage. The transverse deformation of the concrete increases sharply, and the steel tube changes from mainly bearing longitudinal pressure to mainly bearing circumferential tension (i.e., providing constraining force for the concrete). With the increase in the constraining force, the bearing capacity of the internal concrete increases, compensating for the reduction in the longitudinal bearing capacity of the steel tube. Finally, the CFST column enters its limit state when the sum of the pressures that the steel tube and concrete can withstand reaches its maximum value.

**Figure 11:**
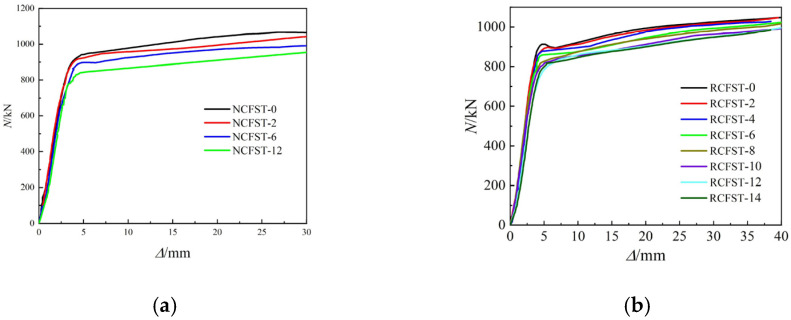
The load–displacement curve: (**a**) NCFST columns; (**b**) RCFST columns.

The axial load–displacement (N-*Δ*) curves for NCFST columns and RCFST columns after different degrees of corrosion are shown in Figure 11, respectively. As can be seen from the figures, the curves are mainly divided into three stages: elastic, elastoplastic, and strengthening, without a descending branch. This is mainly due to the fact that the hoop coefficient is greater than 1.0 and the steel tube has a stronger restraint effect on the concrete [5,6,7]. Therefore, the circular steel tube concrete short column specimens have a significant reinforcing phase and good ductility in the later stage. The trend of the curves is basically the same after experiencing different degrees of corrosion. With the increase in the corrosion degree, the peak load gradually decreases, and the ductility decreases [7].

**Figure 12:**
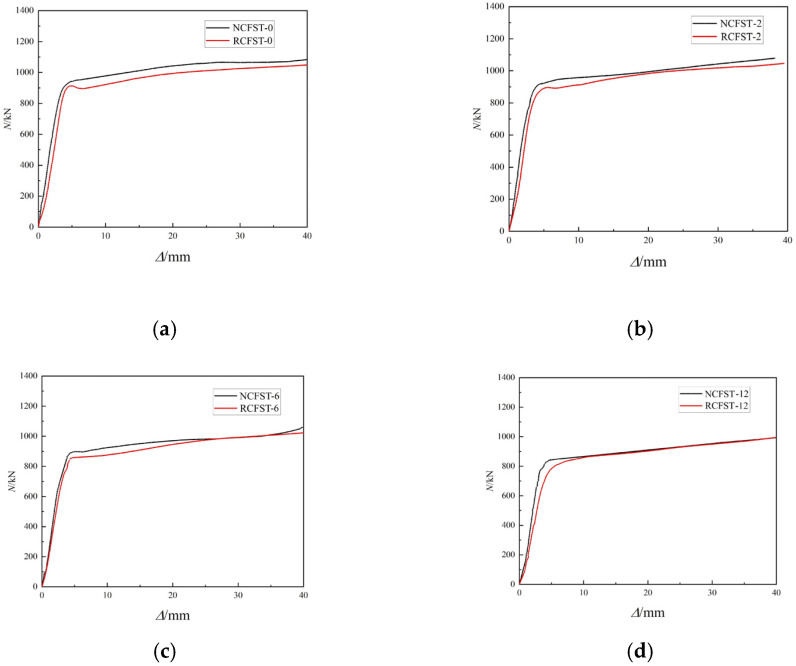
The load–displacement curve type of specimens after different corrosion degree: (**a**) 0%; (**b**) 2% (**c**) 6%; (**d**) 12%.

Figure 12 shows the axial load–displacement (N-*Δ*) curves for NCFST columns and RCFST columns at the same corrosion degree. From Figure 12, it can be seen that the stiffness and bearing capacity of RCFST columns decrease compared with NCFST columns. The addition of recycled aggregates makes the core concrete lower in strength. According to Figure 11, the influence of recycled concrete is significant only in the case of zero corrosion and becomes negligible at higher corrosion levels. At zero corrosion, recycled concrete in RCFST short columns, with old adhesive mortar and possible micro-cracks, has distinct properties that lead to differences in mechanical behavior compared to natural concrete, thus playing a more noticeable role in the columns’ structural response. As corrosion level rises, the steel tube degrades significantly, reducing its cross-sectional area, weakening mechanical properties, introducing stress concentrations, and becoming the dominant load-bearing factor that affects load-carrying and deformation capacities. Moreover, corrosion changes the bond between the steel tube and concrete core, further highlighting the steel tube’s degradation, so the influence of recycled concrete is overshadowed and becomes negligible at higher corrosion levels.

### 3.3. Stiffness

In this test, the axial compressive stiffness was determined using the transformed-section method. The axial stiffness is calculated using the following formula [26]:

(3) $$K_{\mathrm{sc}}=\frac{A_{\mathrm{sc}}E_{\mathrm{sc}}}{L}$$

(4) $$E_{sc}=1.3k_{E}f_{sc}$$

(5) $$f_{sc}=(1.212+B\theta +C\theta^{2})f_{c}$$

(6) $$B=0.1759f_{y}/235+0.974$$

(7) $$C=-0.1028f_{ck}/20+0.0309$$

(8) $$\alpha_{sc}=\frac{A_{s}}{A_{c}}$$

(9) $$\theta =\alpha_{sc}\frac{f_{y}}{f_{c}}$$

The concrete-filled steel tubular (CFST) short column is affected by corrosion, resulting in the thinning of the outer steel tube. The cross-sectional area of the corroded specimen is calculated based on weight conversion. The calculation formula is presented as follows:

(10) $$A_{sc}=\frac{m_{i}}{\rho \cdot h}$$

where the following applies: $K_{sc}$ is the axial stiffness. $E_{sc}$ is the modulus of elasticity of RCFST column. $k_{E}$ is axial compression modulus conversion factor. $f_{sc}$ is design value of compressive strength of RCFST column. $A_{s}$ and $A_{c}$ are the areas of the steel tube and the concrete inside the tube, respectively. $A_{sc}$ is the areas of the RCFST column. $\alpha_{sc}$ is the steel content of RCFST column. θ is the hoop coefficient. $f_{y}$ and $f_{c}$ are the compressive strength characteristic values for steel tube and concrete, respectively. B and C are both influence coefficient of cross-sectional shape on hoop effect.

**Figure 13:**
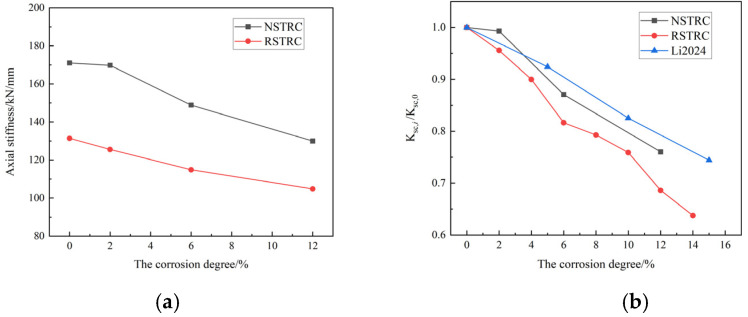
The stiffness of columns versus the corrosion degree: (**a**) Axial stiffness calculation value; (**b**) Relative axial compression stiffness variation [28].

Figure 13a shows the graph of axial compressive stiffness as a function of the corrosion rate, while Figure 13b depicts a graph of the ratio of the axial compressive stiffness of the specimen before and after corrosion ($K_{sc,i}/K_{sc,0}$) versus the corrosion degree. Under uncorroded conditions, the stiffness of the RCFCST column decreased by 23.5% compared with that of the NCFCST column. This is consistent with the findings of Yang [10]. This is also consistent with the findings of others. Yang [10] found that the stiffness of concrete-filled steel tube short columns with recycled aggregates completely replacing natural aggregates was reduced by 21%. This was because the addition of recycled aggregate caused the modulus of elasticity of the concrete to decrease. As can be seen from Figure 13, with the increase in the corrosion degree, the axial compression stiffness gradually decreases. This is mainly due to the reduction in the cross-sectional area of the specimens after corrosion, leading to a decrease in the confinement effect of the steel tube. As the degree of corrosion increases, the axial stiffness of RCFST columns decreases faster than that of NCFST columns. Take the corrosion degree of 12% specimens as an example; $K_{\mathrm{sc}}$ was reduced by 23.5 and 31.1% for the NCFST and RCFST short columns, respectively. Initial defects in recycled concrete are more likely to spread under the penetration of corrosive media, forming through cracks that weaken the constraint effect on steel tubes and accelerate overall stiffness degradation [27]. Figure 13b reveals that there is a downward-trending pattern in the axial compressive stiffness of both NCFST and RCFST samples as the corrosion degree escalates, a phenomenon that is consistent with the experimental results obtained by Li [28]. Due to the fact that the specimen utilized in the experiment had a comparatively large aspect ratio, its axial compressive stiffness underwent a more pronounced and accelerated decline.

### 3.4. Ductility Index

Ductility is an important indicator for evaluating the deformation capacity of structures. In order to analyze the effect of corrosion degree on the ductility of specimens, a ductility index (DI) is defined, as shown in Equation (11).

(11) $$DI=\frac{\varepsilon_{u}}{\varepsilon_{y}}$$

where $\varepsilon_{u}$ is the corresponding strain at the peak load. $\varepsilon_{y}$ is the corresponding strain at the initial yield point. The position of the initial yield point is determined using the equivalent energy method.

**Figure 14:**
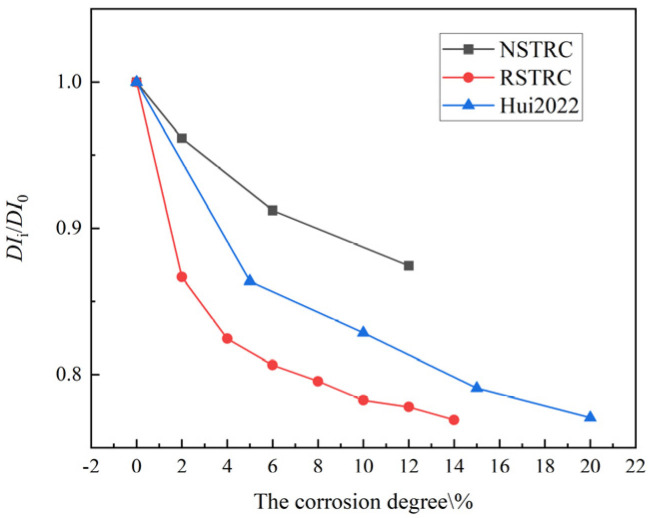
The *DI_i_*/*DI*_0_ of columns versus the corrosion degree [27].

The variation in $DI_{i}/DI_{0}$ (ductility coefficient after different corrosion degrees/ductility coefficient of uncorroded specimens) with respect to the corrosion degree is shown in Figure 14. Corrosion has a significant impact on the ductility of steel-reinforced concrete specimens. As the corrosion degree increases from 0 to 2, 6, and 12%, the ductility coefficients of NCFST columns and RCFST columns decrease by 4.7, 8.7, and 13.6% and 13.4, 19.4, and 22.3%, respectively. By comparing the ductility changes in NCFST columns and RCFST columns at the same corrosion degree, it can be observed that their ductility reduction trends are similar, but the RCFST columns exhibits a higher rate of reduction. Hui [27] found that the ductility coefficient of the RCFST column with 10% corrosion was reduced by 17.15%, and the ductility coefficient of the RCFST column with 10% corrosion was reduced by 21.8% in this test. This is mainly due to the difference in the substitution rate of recycled coarse aggregate, which is 50 and 100%, respectively, between Hui and the samples in this experiment. Although the ductility of the concrete-filled steel tube short columns is increased by the presence of the external steel tube, corrosion reduces the wall thickness of the tubes, and the restraining effect of the external steel tube on the core concrete is reduced.

### 3.5. Bearing Capacity

Currently, there is no specific code for the calculation of the bearing capacity of RCFST columns. The calculation method for the bearing capacity of NCFST columns is often used as a reference. The theoretical basis for the calculation formula of the bearing capacity of NCFST columns can be roughly divided into two types. One type is the superposition theory that simply adds the steel tube and the core concrete without considering their interaction [29,30]. The other type is the unified theory, which considers the interaction between the steel tube and the core concrete [26]. A degradation model for the bearing capacity of recycled steel-reinforced concrete after corrosion is proposed based on the unified theory in the code GB50936-2014 [26]. The model is represented by the following equation:

(12) $$N_{ue}=0.96a_{v}N_{0}$$

(13) $$N_{0}=A_{sc}f_{sc}$$

(14) $$NCFST\;column:\;\;a_{v}=0.987-0.0085\eta \;\;R^{2}=0.99$$

where $N_{ue}$ is the compressive bearing capacity of RCFST column. $N_{0}$ is the compressive bearing capacity of uncorroded specimens. $f_{sc}$ is design value of compressive strength of RCFST column. $A_{s}\;\mathrm{and}\;A_{c}$ are the areas of the steel tube and the concrete inside the tube, respectively. $A_{sc}$ is the areas of the RCFST column. $\alpha_{sc}$ is the steel content of RCFST column. θ is the hoop coefficient. $f\;\mathrm{and}\;f_{c}$ are the compressive strength design values for steel tube and concrete, respectively. $f_{y}\;\mathrm{and}\;f_{ck}$ are the compressive strength characteristic values for steel tube and concrete, respectively. B and C are both influence coefficient of cross-sectional shape on hoop effect. $a_{\nu }$ is the influence coefficient of corrosion degree on bearing capacity. η is the corrosion degree (%). 0.96 is the reduction coefficient of RCFST column.

**Figure 15:**
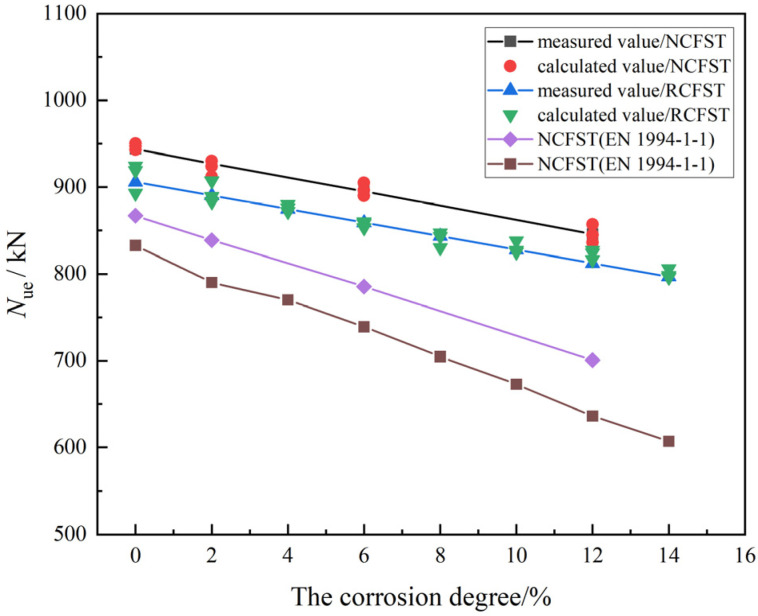
Comparison of measured bearing capacity and calculated bearing capacity [30].

The bearing capacity of both the NCFST and RCFST columns decreased with an increasing corrosion degree. There are three main reasons for this phenomenon. One is that the yield strength and tensile strength of the steel tube are reduced due to corrosion. The second is that the wall thickness of the steel tube becomes thinner due to corrosion, and the restraining effect of the steel tube on the concrete is weakened. The last factor is that the bond performance between concrete and steel tube is reduced due to corrosion. In addition, it can be seen from Figure 15 that the bearing capacity of RCFST columns is about 20–30 kN lower than that of NCFST columns for the same corrosion degree. This is mainly due to the fact that the mechanical properties of recycled concrete are poorer than natural concrete for the same mix ratio. The incorporation of recycled aggregates resulted in deterioration of both strength and ductility of the concrete. As shown in Figure 15, the calculation results of EC4 yield a more conservative estimate of the axial capacity of the specimens, primarily due to the fact that this specification fails to account for the confinement effect exerted by the steel tubes on the core concrete, which would otherwise enhance the load-bearing capacity of the composite structure.

**Figure 16:**
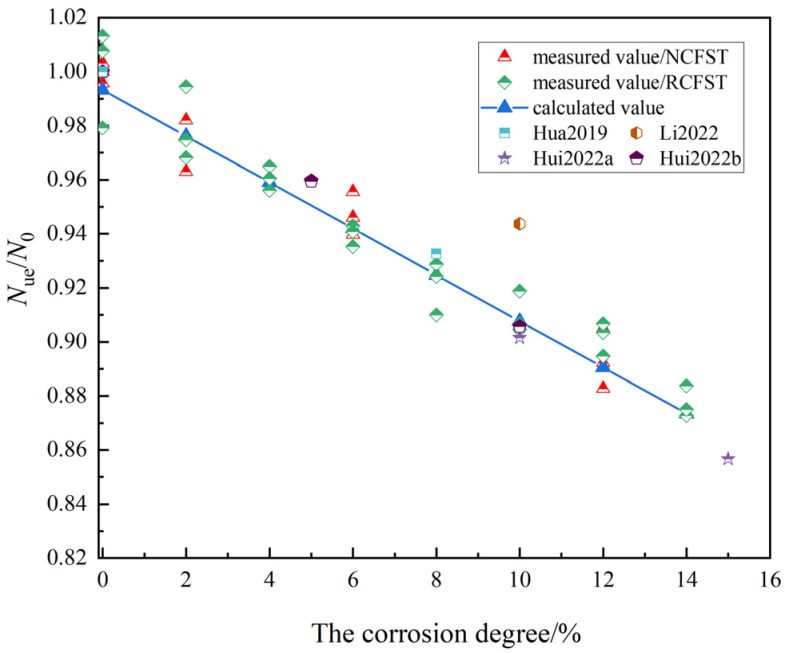
Validation of ultimate load capacity calculation models [14,16,19,27].

As shown in Figure 16, the model calculations fit well with the experimental data of some scholars. The ultimate bearing capacity of RCFST columns with a corrosion degree of 10% decreases by approximately 9.4%, which is in close proximity to the experimental results obtained by some scholars [13,27]. In contrast, the relatively small reduction in bearing capacity in Li’s case is due to local corrosion [16]. Due to the small number of corrosion rate and concrete substitution rate groups in this test, the calculation model of this paper involved certain limitations.

## 4. Conclusions

In this paper, the effects of the corrosion degree on the axial compressive properties of circular concrete-filled steel tubular columns completely replaced with recycled aggregates were investigated through experimental and theoretical analysis. The main conclusions are as follows:

1. The specimens were all damaged due to localized buckling deformation. The damage patterns of both NCFST and RCFST columns are in the form of a waist drum, mainly due to the constraint coefficient being greater than 1.0. The damage mode is less affected by the corrosion degree for larger constraint coefficients.
2. With the increase in the corrosion degree, the bearing capacity of the specimens was reduced, and the stiffness and ductility were decreased to different degrees. The ductility coefficient and stiffness of RCFST are significantly lower than those of NCFST. However, with the increase in the corrosion degree, the ductility coefficient and stiffness reduction law of RCFST and NCFST are roughly similar.
3. The ductility is more affected by the corrosion degree than the ultimate bearing capacity for larger constraint coefficients.
4. The axial compressive load capacity calculation model of concrete-filled steel tubular columns considering the corrosion degree of a steel tube was proposed based on the unified theory, and the fitting results are good. Furthermore, the investigation was limited to the effects of different corrosion degrees and concrete types on the axial compression performance of circular concrete-filled steel tubular columns. There are many areas worthy of further exploration in the future, such as the effects of different corrosion times and different steel grades on the axial compression performance of RCFST columns under the same corrosion degree.

## Figures and Tables

**Table 1:** The parameters and results of specimens.

Column Number	*D* ^2^ (mm)	*L* ^3^ (mm)	*t* ^4^ (mm)	*α_s_* ^5^	*η*_t_ ^6^ (%)	*η*_a_ ^7^ (%)	*T* ^8^ (h)	*N_ue_* ^9^
NCFST-0-1 ^1^	108	300	4	0.166	0	0.1	0	947
NCFST-0-2	108	300	4	0.166	0	0.15	0	943
NCFST-0-3	108	300	4	0.166	0	0.4	0	950
NCFST-2-1	108	300	4	0.166	2	2.0	58	912
NCFST-2-2	108	300	4	0.166	2	2.2	58	930
NCFST-2-3	108	300	4	0.166	2	2.1	58	924
NCFST-6-1	108	300	4	0.166	6	5.5	173	905
NCFST-6-2	108	300	4	0.166	6	6.2	173	890
NCFST-6-3	108	300	4	0.166	6	5.7	173	896
NCFST-12-1	108	300	4	0.166	12	11.8	347	857
NCFST-12-2	108	300	4	0.166	12	12.3	347	836
NCFST-12-3	108	300	4	0.166	12	11.4	347	845
RCFST-0-1	108	300	4	0.166	0	0.5	0	924
RCFST-0-2	108	300	4	0.166	0	0.6	0	919
RCFST-0-3	108	300	4	0.166	0	0.7	0	893
RCFST-2-1	108	300	4	0.166	2	2.6	58	907
RCFST-2-2	108	300	4	0.166	2	2.4	58	889
RCFST-2-3	108	300	4	0.166	2	2.8	58	883
RCFST-4-1	108	300	4	0.166	4	4.8	116	880
RCFST-4-2	108	300	4	0.166	4	4.6	116	872
RCFST-4-3	108	300	4	0.166	4	5.1	116	876
RCFST-6-1	108	300	4	0.166	6	6.3	173	860
RCFST-6-2	108	300	4	0.166	6	6.5	173	853
RCFST-6-3	108	300	4	0.166	6	6.1	173	858
RCFST-8-1	108	300	4	0.166	8	8.5	231	847
RCFST-8-2	108	300	4	0.166	8	8.3	231	843
RCFST-8-3	108	300	4	0.166	8	8.1	231	830
RCFST-10-1	108	300	4	0.166	10	9.8	289	838
RCFST-10-2	108	300	4	0.166	10	10.2	289	827
RCFST-10-3	108	300	4	0.166	10	9.7	289	825
RCFST-12-1	108	300	4	0.166	12	12.1	347	824
RCFST-12-2	108	300	4	0.166	12	11.6	347	816
RCFST-12-3	108	300	4	0.166	12	12.9	347	827
RCFST-14-1	108	300	4	0.166	14	14.3	405	796
RCFST-14-2	108	300	4	0.166	14	14.1	405	806
RCFST-14-3	108	300	4	0.166	14	13.9	405	798

^1^ NCFST-0-1 = natural circular concrete-filled steel tubular-corrosion degree-number; ^2^ *D* = the cross-sectional diameter of the specimen; ^3^ *L* = the height of the specimen; ^4^ *t* = the wall thickness of the specimen; ^5^ *α_s_* = the ratio of the cross-sectional area of the steel tube to the cross-sectional area of the concrete; ^6^ *η*_t_ = the theoretical corrosion degree; ^7^ *η*_a_ = the actual corrosion degree; ^8^ *T* = corrosion duration; ^9^ *N_ue_* = ultimate load-bearing capacity.

**Table 2:** The basic properties of recycled coarse aggregates.

No.	Crush Value (%)	Water Absorption (%)	Apparent Density
NCFST-0-1	108	300	4
NCFST-0-2	108	300	4

**Table 3:** Mix proportion of concrete.

No.	Cement (kg/m^3^)	FA (kg/m^3^)	NCA (kg/m^3^)	RCA (kg/m^3^)	Water (kg/m^3^)
RCFST	418	610	0	1184	188
NCFST	418	300	4		

**Table 4:** The properties of steel tube tensile specimens.

No.	*t*_s_ ^1^ (mm)	*η*_t_ ^2^ (%)	*η*_a_ ^3^ (%)	*E*_s_ ^4^ (MPa)	*f*_y_ ^5^ (MPa)	*f*_u_ ^6^ (MPa)	*e* ^7^ (%)
L-0	4	0	0	1.92 × 10^5^	479.2	612.6	32
L-2%	4	2	2.4	1.92 × 10^5^	486.1	611.5	34
L-4%	4	4	4.4	1.91 × 10^5^	472.9	605.2	27
L-6%	4	6	6.3	1.90 × 10^5^	467.9	602.4	26
L-8%	4	8	8.3	1.89 × 10^5^	454.1	563.2	23
L-10%	4	10	10.4	1.88 × 10^5^	451.2	558.1	23
L-12%	4	12	12.5	1.88 × 10^5^	439.5	561.7	23
L-14%	4	14	16.6	1.87 × 10^5^	408.1	450.1	17

^1^ *t*_s_ = the thickness of the tensile specimens; ^2^ *η*_t_ = the theoretical corrosion degree; ^3^ *η*_a_ = the actual corrosion degree; ^4^ *E*_s_ is the elastic modulus; ^5^ *f*_y_ is the yield strength; ^6^ *f*_u_ is the ultimate strength; ^7^ *e* is the Elongation.

## Data Availability

The original contributions presented in this study are included in the article. Further inquiries can be directed to the corresponding author.

## Abbreviations

The following abbreviations are used in this manuscript:

RCFST	Recycled circular concrete-filled steel tubular
NCFST	Natural circular concrete-filled steel tubular
FA	Fine aggregate
NCA	Natural coarse aggregate
RCA	Recycled coarse aggregate
